# The claudin–transcription factor signaling pathway

**DOI:** 10.1080/21688370.2021.1908109

**Published:** 2021-04-27

**Authors:** Kotaro Sugimoto, Hideki Chiba

**Affiliations:** Department of Basic Pathology, Fukushima Medical University School of Medicine, Fukushima, Japan

**Keywords:** Tight junction, CLDN, cell adhesion signal, signal transduction, PI3K, AKT, nuclear receptor, Src-family kinase, transcription factors

## Abstract

Claudins (CLDNs) represent major transmembrane proteins of tight junctions and contribute to the barrier function. They also serve as anchors for several signaling proteins, but the underlying molecular basis has yet to be established. The present review covers the recent progress in our understanding of the CLDN signaling pathway in health and disease. We discuss the functional relevance of phosphotyrosine motifs in the C-terminal cytoplasmic domain of CLDNs and define mutual regulation between CLDNs and Src-family kinases (SFKs). In addition, we focus on the crosstalk between CLDN and transcription factor signaling. We also describe how aberrant CLDN–transcription factor signaling promotes or inhibits cancer progression. We propose that a link between various cell adhesion molecules and transcription factors coordinates a range of physiological and pathological events via activation or suppression of target genes.

## Introduction

The claudin (CLDN) family is capable of forming tight-junction strands and thereby represents the backbone of tight junctions in vertebrate epithelial and endothelial cells, as well as in other types of cells.^**1,2**^ It is composed of 27 members in mammals, and a specific combination of CLDNs is expressed in a given cell/tissue type. CLDNs are tetraspan transmembrane proteins that include two extracellular loops (EC1 and EC2) and N- and C-terminal cytoplasmic domains. The CLDN-EC1 creates paracellular barriers or pores for selective ions and solutes,^**3–7**^ whereas both CLDN-EC1 and CLDN-EC2 contribute to *cis*- and *trans*-interactions between CLDNs.^**7–11**^ On the other hand, the C-terminal cytoplasmic domain of many CLDN subtypes contains specific sequence motifs such as PDZ domain-binding motifs and phosphorylation consensus sites^**5, 12–16**^ and receives or propagates a magnitude of intracellular signals as platforms; however, it remains poorly defined how CLDN signaling reaches the nucleus and regulates gene expression.^**17**^

CLDNs are absolutely required for human health, and their dysregulations are involved in the pathogenesis of diverse diseases.^**4-6,18-16**^ For instance, it is well known that mutations in several *CLDN* genes cause various human hereditary diseases. In addition, CLDNs frequently show aberrant expression and/or localization in a wide variety of cancers, resulting in either promotion or repression of tumor progression, most probably by dysregulated CLDN signaling.^**19–25,22,26–32**^ Moreover, recent studies have established the region-selective CLDN5 breakdown in brain disorders, such as schizophrenia, depression, Alzheimer’s disease, and multiple sclerosis.^**33–39**^ In this respect, we and others have previously reported how endothelial CLDN5 expression is regionally disrupted in these psychiatric disorders.^**40,41**^

In the current review, we focus on the link between CLDN and transcription factor signaling because it is theoretically attributed to the organization of a broad range of cellular processes, including cell growth, survival, differentiation, polarity, migration, and metabolism, via regulation of the expression of corresponding target genes. We also discuss aberrant CLDN–transcription factor signaling in cancer. We do not describe recent progress in our understanding of numerous aspects of other tight-junction players, such as junctional adhesion molecules (JAMs), tight junction-associated MARVEL domain-containing proteins (TAMPs: occludin, tricellulin, and MarvelD3), and the ZO family of scaffolding proteins.^**42–55**^

### The CLDN/SFK/PI3K/AKT/transcription factor signaling cascade

We previously reported that retinoid X receptor α (RXRα)/retinoic acid receptor γ (RARγ) and another member of the nuclear receptor superfamily, hepatocyte nuclear factor 4α (HNF4α), trigger the formation of mature cell–cell junctions and microvilli, expression of tight-junction markers (CLDN6, CLDN7, occludin, and ZO-1α+ variant) and a microvilli marker (ezrin/radixin/moesin-binding phosphoprotein 50 [EBP50]), as well as morphological differentiation into epithelial cells from stem cells.^56–59^ These effects are incredibly similar to the CLDN6-triggered ones,^60^ implying a possible crosstalk between them. Along this line, we have recently identified the CLDN–transcription factor signaling pathway.^61^

## Reciprocal regulation between CLDNs and SFKs

**Figure 1.**
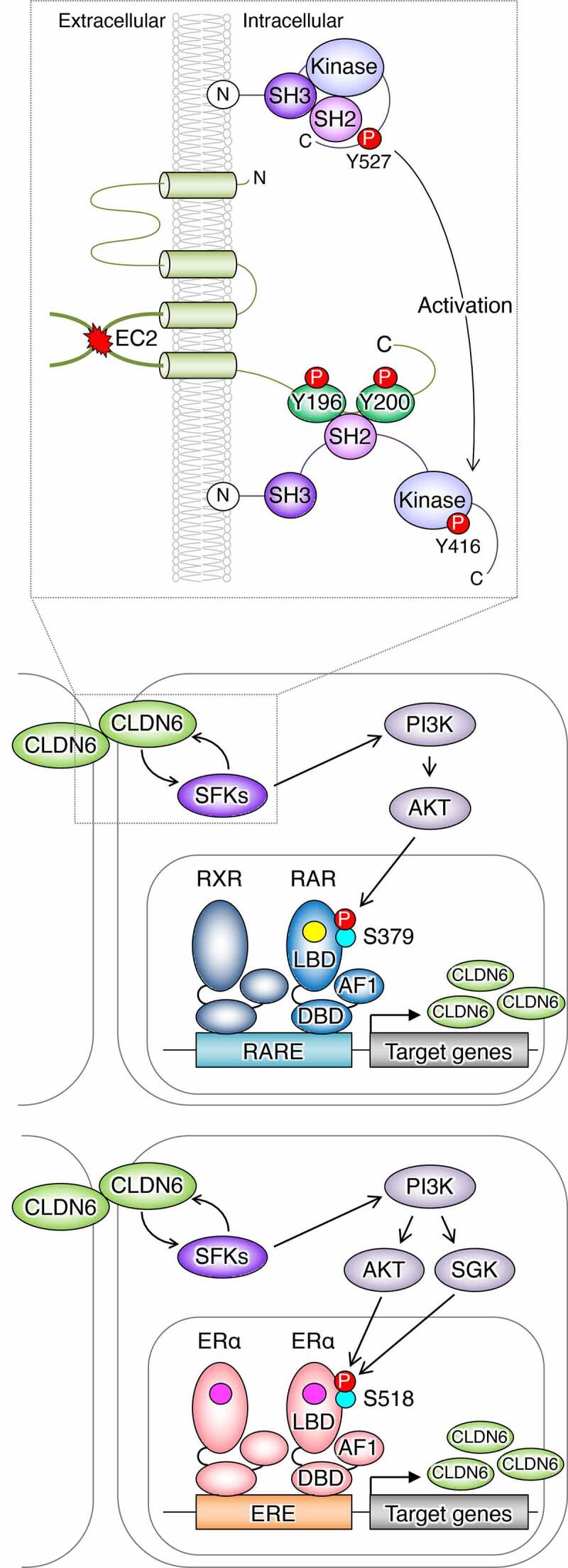
Schematic model for regulation of the nuclear receptor activity by the CLDN–adhesion signaling. The schema is modified from that reported previously (Sugimoto et al., 2019). SH2/3: Src homology 2/3 domain; Kinase: kinase domain; AF1: activation function-1; DBD: DNA-binding domain; LBD: ligand-binding domain; RARE: retinoic acid response element; ERE: estrogen response element; RA: yellow circle; estrogen: pink circle

First, we showed, by using the corresponding deletion mutants, that the CLDN6–adhesion signal is transduced through the EC2 domain and the first half C-terminal cytoplasmic domains but not through the EC1 domain (Figure 1). Second, we paid attention to the four tyrosine residues in the C-terminal domain of CLDN6, which are completely conserved among vertebrates, and revealed that Y196/200, but not Y213/218, are definitely required for CLDN6-signaling ability. Third, we disclosed that CLDN6 recruits and activates Src-family kinases (SFKs) in EC2-dependent and Y196/200-dependent manners, and SFKs in turn phosphorylate CLDN6 at Y196/200 in an EC2-dependent fashion. The functional relevance of the EC2 in the CLDN6–adhesion signal was also supported by using the C-terminal half of *Clostridium perfringens* enterotoxin (C-CPE).^60,61^ Moreover, we have identified SFK members associated with CLDN6 and obtained evidence showing that recombinant proteins corresponding to the Src homology 2 (SH2) domains of certain SFKs directly bind to the C-terminal cytoplasmic domain of CLDN6 and the Y196/200-containing peptide (our unpublished results).

**Figure 2.**
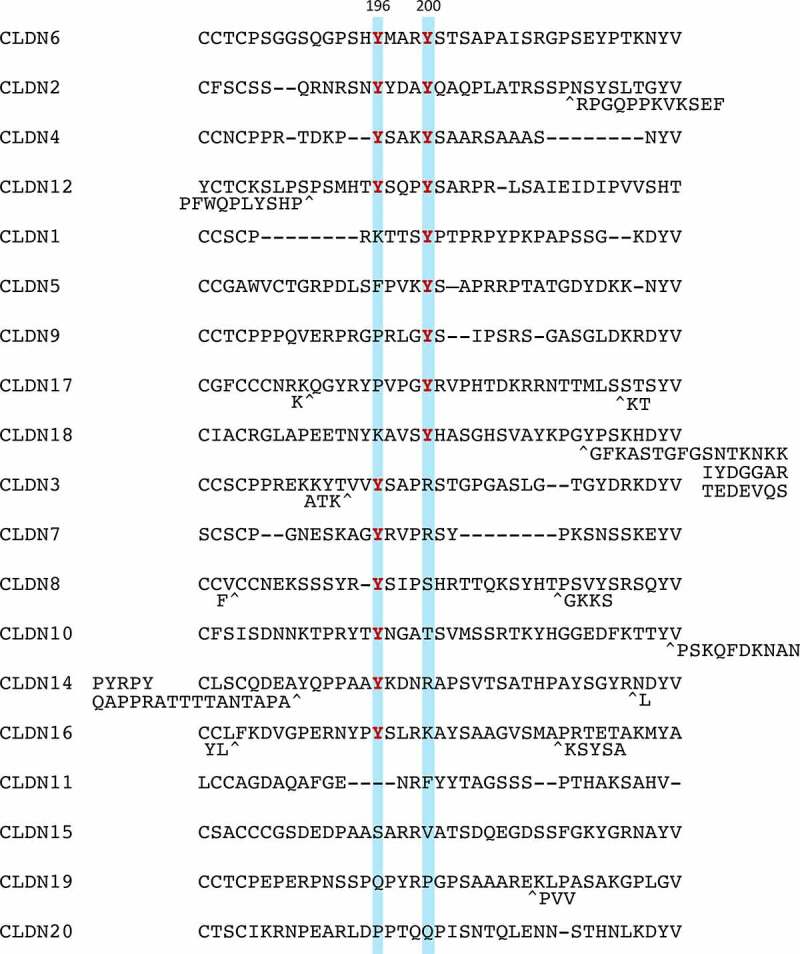
Amino acid sequences of a part of the C-terminal cytoplasmic domain in human CLDN1-20. Tyrosine residues corresponding to CLDN6Y196/200 are highlighted and the conserved ones are indicated in red

**Figure 3.**
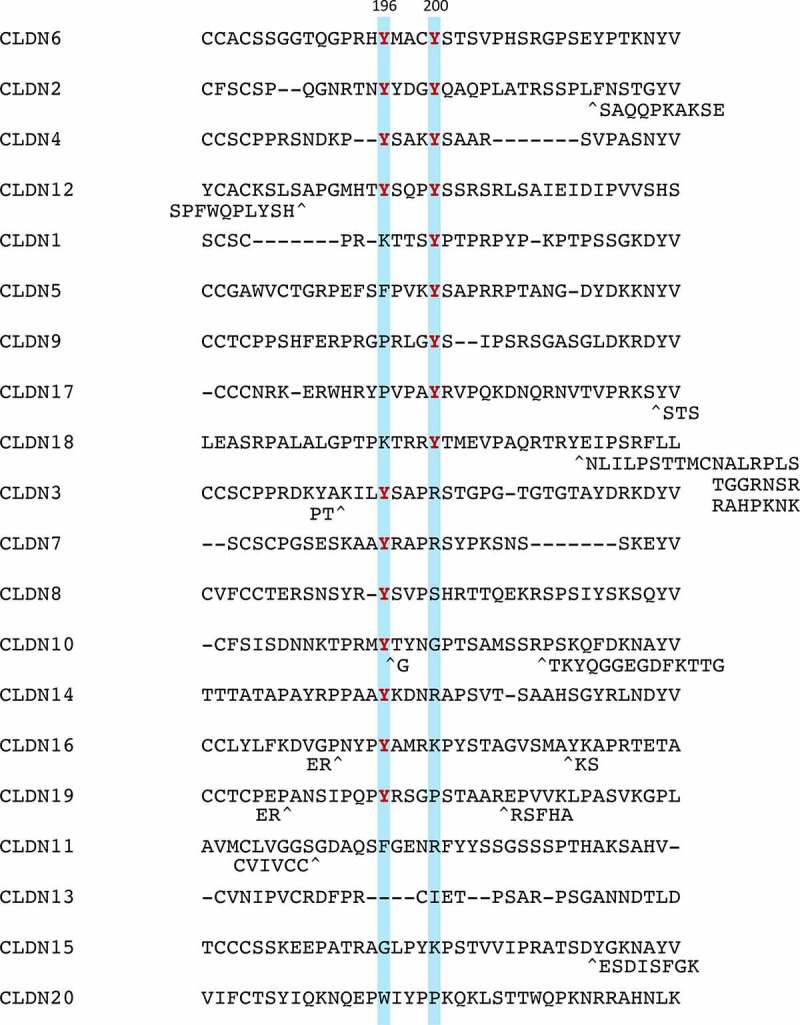
Amino acid sequences of a part of the C-terminal cytoplasmic domain in mouse CLDN1-20. Tyrosine residues corresponding to CLDN6Y196/200 are highlighted and the conserved ones are indicated in red

Through a careful search for amino acid sequence of human and mouse CLDN1–20, both Y196 and Y200 in the C-terminal cytoplasmic domain of CLDN6 are conserved in CLDN2/4/12 (Figures 2 and 3). It is also noteworthy that CLDN6Y196 and CLDN6Y200 are conserved in CLDN3/7/8/10/14/16 and CLDN1/5/9/17/18, respectively.

We have found mutual regulation between certain CLDN subtypes (tyrosine residues corresponding to CLDN6Y200) and SFKs (our unpublished results). In addition, Li et al.^*62*^ have recently reported that CLDN11 is phosphorylated at two adjacent tyrosine residues, which are positioned at different sites from CLDN6Y196/200, as described above, and activates SRC. The C-terminal domain of CLDN1 is also associated with SRC although the involvement of the pY motifs is not determined.^63^ It should also be noted that amino acids around pY, in particular 3–5 residues at the C-terminal side, influence the binding specificity of the SH2 domain.^64–67^ For instance, the C-terminal amino acids of the CLDN6Y200-corresponding tyrosine residue in human and mouse CLDN18 are very different from others; therefore, it does not seem to function as the pY motif. CLDN18 is known to decrease PI3K and AKT activities,^68,69^ suggesting this notion. Further studies are required to determine whether other CLDN subtypes, similar to CLDN6, couple with SFKs via the pY motifs in the C-terminal cytoplasmic domains.

SFKs are known to be activated by several cell–cell and cell–matrix adhesion proteins lacking intrinsic kinase activity, such as E-cadherin, integrins, and cellular prion protein.^62^,^70–76^, In addition, engagement of JAMs, the JAML (junctional adhesion molecule-like) and CAR (coxsackie and adenovirus receptor), stimulates PI3K,^77^which is the major downstream signal of SFKs. Besides, it is well known that certain signaling proteins, which contain the SH2, phosphotyrosine-binding (PTB), Hakai-tyrosine binding (HYB), C2, and pyruvate kinase M2 domains, bind to the pY motifs.^66,67,78–81^ Taken together, these findings strongly suggest that the pY motifs in the C-terminal cytoplasmic domains of diverse cell–cell and cell–matrix adhesion molecules generally serve as the signaling landscapes for SFKs and other pY motif-binding proteins.

## A link between CLDN/SFK and transcription factor signalings

Importantly, the CLDN6/SFK/PI3K/AKT axis targets the AKT-phosphorylation sites in the RARγ and the estrogen receptor α (ERα) and stimulates their activities, thereby regulating the expression of respective target genes. This conclusion was drawn from the following results: (1) the CLDN6-induced cellular events were hindered in three distinct F9:*Rxra^–/–^:Rarg^–/–^:Cldn6* cell lines, despite SFKs being activated; (2) AKT formed a complex with either RXRα/RARγ or ERα; and (3) characterization of F9:*Rxra^–/–^:Rarg^–/–^:Cldn6:iRxra-Rarg2S379A* (hereafter, “*i*” means doxycycline-inducible expression of a give gene) and F9:*Rxra^–/–^:Rarg^–/–^:Cldn6:iRxra-Rarg2S379E* cells, as well as MCF-7:*ESR1S518E* cells, revealed that CLDN6 signaling directs S379 and S518 in mouse RARγ and human ERα, respectively. In addition, CLDN6-provoked RARγS379 phosphorylation in mice resulted in releasing the nuclear receptor corepressor (NCoR) from several retinoic acid response elements (RAREs) of three distinct RA target genes, including Cldn6. Since RXRα/RARγ heterodimer appears to induce Cldn6 gene expression,^56,61^ the positive loop of the CLDN6–RARγ cascade could contribute not only to triggering but also to the maintenance of CLDN6-initiated cellular events. Intriguingly, the AKT-consensus phosphorylation motifs are conserved in 14 of 48 members of human nuclear receptors, implying the biological relevance of this phosphorylation site.

## Aberrant CLDN–transcription factor signaling in cancer

We have recently reported that aberrant CLDN6 expression in endometrial cancer tissues is significantly associated with several clinicopathological variables, such as surgical stage III/IV, histological type, histological grade 3, lymphovascular space involvement, lymph node metastasis, and distant metastasis.^82^ Additionally, we showed that the high CLDN6 expression in endometrial cancer represents an independent prognosis marker, and the 5-y survival rate was approximately 30%, which was one-third of that in the low-expression group.

**Figure 4.**
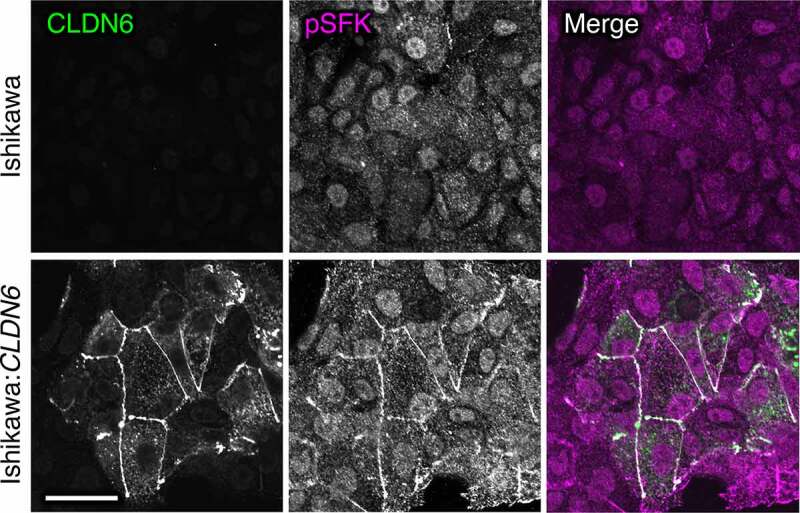
Aberrant SFK activation in human endometrial cancer cells by CLDN6. Scale bar, 20 μm

**Figure 5.**
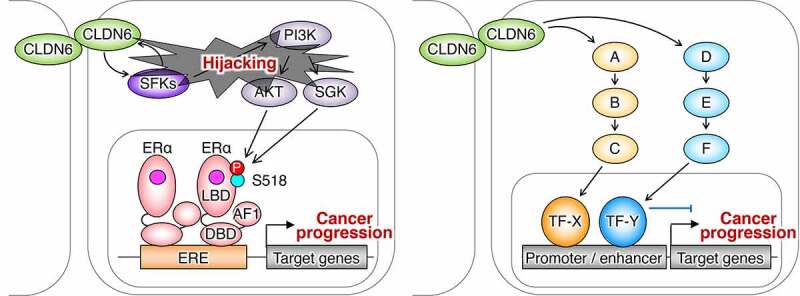
The ERα-dependent and independent CLDN6 signaling in endometrial cancer. A–F indicate signaling proteins. TF, transcription factor

In an additional study, we found that aberrant CLDN6 expression promotes the malignant phenotypes of endometrial cancer *in vitro* and *in vivo* via hijacking the CLDN6–ERα axis.^83^ For instance, we demonstrated that abnormal CLDN6–ERα signaling stimulates not only cell proliferation but also collective cell migration in the leading front of endometrial cancer cells. It is noteworthy that activated SFKs appear to be concentrated at the cell borders together with CLDN6 in Ishikawa:*CLDN6* cells but not in parental Ishikawa cells (Figure 4). The EC2 domain and Y196/200 of CLDN6 were required to recruit and activate SFKs and to stimulate malignant phenotypes of endometrial cancer cells. In addition, the CLDN6/SFK/PI3K pathway propagates both AKT and serum- and glucocorticoid-regulated kinase (SGK), which share a high degree of homology and the same consensus phosphorylation motif,^84^ resulting in targeting S518 in human ERα and activating target genes in a ligand-independent manner. Furthermore, RNAseq and RT-qPCR analyses indicated the presence of not only ERα-dependent but also ERα-independent CLDN6 signaling (Figure 5). The identification of this machinery highlights the regulation of transcription factor activity by cell adhesion to advance tumor progression.

Another issue that should be mentioned is other abnormal CLDN–transcription factor signaling in health and disease. The CLDN18/Yes-associated protein (YAP) pathway regulates the homeostasis of normal lung stem and progenitor cells, and its deficiency promotes tumorigenesis and progression of lung and gastric adenocarcinoma.^69,85–87^ By contrast, CLDN2 activates YAP, leading to self-renewal of human colorectal cancer stem-like cells.^88^ Since CLDN2 has two conserved pY in the C-terminal cytoplasmic domain as described above, it should also be verified whether CLDN2/SFK signaling is involved in the self-renewal of various cancer stem-like cells.

## Conclusions and future directions

The work discussed in the present review highlights the CLDN–transcription factor signaling pathway, notably the CLDN/SFK/PI3K/AKT/nuclear receptor cascade. Cell–cell and cell–matrix adhesion molecules are indispensable not only for proper tissue integrity but also for signaling properties that coordinate a wide range of cell behaviors. In other words, appropriate tissue formation connected by various cell adhesion proteins should be pre-requested for normal cell-adhesion signal. Since cell–cell and cell–matrix adhesion proteins are broadly expressed in distinct cell types, we propose that various combinations of cell adhesion molecules and transcription factors coordinate diverse physiological and pathological processes, including cancer. The cell-adhesion signals most probably lead to posttranslational modification of transcription factors, thereby regulating their activities. In future, it would be interesting to generalize the cell adhesion–transcription factor signaling pathway in health and disease.

## References

[1] Furuse M, Fujita K, Hiiragi T, Fujimoto K, Tsukita S. Claudin-1 and −2: novel integral membrane proteins localizing at tight junctions with no sequence similarity to occludin. J Cell Biol. 1998;141(7):1–11. doi:10.1083/jcb.141.7.1539. 9647647 PMC2132999

[2] Furuse M, Sasaki H, Fujimoto K, Tsukita S. A single gene product, claudin-1 or −2, reconstitutes tight junction strands and recruits occludin in fibroblasts. J Cell Biol. 1998;143(2):391–401. doi:10.1083/jcb.143.2.391. 9786950 PMC2132845

[3] Furuse M, Tsukita S. Claudins in occluding junctions of humans and flies. Trends Cell Biol. 2006;16(4):181–188. doi:10.1016/j.tcb.2006.02.006. 16537104

[4] Van Itallie CM, Anderson JM. Claudins and epithelial paracellular transport. Annu Rev Physiol. 2006;68(1):403–429. doi:10.1146/annurev.physiol.68.040104.131404. 16460278

[5] Chiba H, Osanai M, Murata M, Kojima T, Sawada N. Transmembrane proteins of tight junctions. Biochim Biophys Acta. 2008;1778(3):588–600. doi:10.1016/j.bbamem.2007.08.017. 17916321

[6] Zihni C, Mills C, Matter K, Balda MS. Tight junctions: from simple barriers to multifunctional molecular gates. Nat Rev Mol Cell Biol. 2016;17(9):564–580. doi:10.1038/nrm.2016.80. 27353478

[7] Tsukita S, Tanaka H, Tamura TA. The claudins: from tight junctions to biological systems. Trends Biochem Sci. 2019;44(2):141–152. doi:10.1016/j.tibs.2018.09.008. 30665499

[8] Krause G, Winkler L, Mueller SL, Haseloff RF, Piontek J, Blasig IE. Structure and function of claudins. Biochim Biophys Acta. 2008;1778(3):631–645. doi:10.1016/j.bbamem.2007.10.018. 18036336

[9] Piontek J, Winkler L, Wolburg H, Müller SL, Zuleger N, Piehl C, Wiesner B, Krause G, Blasig IE. Formation of tight junction: determinants of homophilic interaction between classic claudins. FASEB J. 2008;22(1):146–158. doi:10.1096/fj.07-8319com. 17761522

[10] Suzuki H, Nishizawa T, Tani K, Yamazaki Y, Tamura A, Ishitani R, Dohmae N, Tsukita S, Nureki O, Fujiyoshi Y. Crystal structure of a claudin provides insight into the architecture of tight junctions. Science. 2014;344(6181):304–307. doi:10.1126/science.1248571. 24744376

[11] Haseloff RF, Piontek J, Blasig IE. Chapter 5 – the investigation of cis- and trans-interactions between claudins. In: Yu AS L editor. Current topics in membranes. Academic Press, USA; 2010. p. 97–112.

[12] Itoh M, Furuse M, Morita K, Kubota K, Saitou M, Tsukita S. Direct binding of three tight junction-associated MAGUKs, ZO-1, ZO-2, and ZO-3, with the COOH termini of claudins. J Cell Biol. 1999;147(6):1351–1363. doi:10.1083/jcb.147.6.1351. 10601346 PMC2168087

[13] Tsukita S, Furuse M, Itoh M. Multifunctional strands in tight junctions. Nat Rev Mol Cell Biol. 2001;2(4):285–293. doi:10.1038/35067088. 11283726

[14] González-Mariscal L, Garay E, Quirós M. Chapter 6 – regulation of claudins by posttranslational modifications and cell-signaling cascades. In: Yu AS L editor. Current topics in membranes. Academic Press, USA; 2010. p. 113–150.

[15] Van Itallie CM, Anderson JM. Phosphorylation of tight junction transmembrane proteins: many sites, much to do. Tissue Barriers. 2018;6(1):e1382671. doi:10.1080/21688370.2017.1382671. 29083946 PMC5823547

[16] Tabariès S, McNulty A, Ouellet V, Annis MG, Dessureault M, Vinette M, Hachem Y, Lavoie B, Omeroglu A, Simon H-G, et al. Afadin cooperates with claudin-2 to promote breast cancer metastasis. Genes Dev. 2019;33(3–4):180–193. doi:10.1101/gad.319194.118. 30692208 PMC6362814

[17] Cavallaro U, Dejana E. Adhesion molecule signalling: not always a sticky business. Nat Rev Mol Cell Biol. 2011;12(3):189–197. doi:10.1038/nrm3068. 21346732

[18] Günzel D, Yu ASL. Claudins and the modulation of tight junction permeability. Physiol Rev. 2013;93(2):525–569. doi:10.1152/physrev.00019.2012. 23589827 PMC3768107

[19] Sawada N. Tight junction-related human diseases. Pathol Int. 2013;63(1):1–12. doi:10.1111/pin.12021. 23356220 PMC7168075

[20] Barmeyer C, Schulzke JD, Fromm M. Claudin-related intestinal diseases. Semin Cell Dev Biol. 2015;42:30–38. doi:10.1016/j.semcdb.2015.05.006. 25999319

[21] Tawar RG, Colpitts CC, Lupberger J, El-Saghire H, Zeisel MB, Baumert TF. Claudins and pathogenesis of viral infection. Semin Cell Dev Biol. 2015;42:39–46. doi:10.1016/j.semcdb.2015.04.011. 25960372

[22] Zeisel MB, Dhawan P, Baumert TF. Tight junction proteins in gastrointestinal and liver disease. Gut. 2019;68(3):547–561. doi:10.1136/gutjnl-2018-316906. 30297438 PMC6453741

[23] Culemann S, Grüneboom A, Nicolás-Ávila JÁ, Weidner D, Lämmle KF, Rothe T, Quintana JA, Kirchner P, Krljanac B, Eberhardt M, et al. Locally renewing resident synovial macrophages provide a protective barrier for the joint. Nature. 2019;572(7771):670–675. doi:10.1038/s41586-019-1471-1. 31391580 PMC6805223

[24] Zhu Z, Yu J, Lin W, Tang H, Zhang W, Lu B. Molecular hydrogen accelerates the reversal of acute obstructive cholangitis‑induced liver dysfunction by restoring gap and tight junctions. Mol Med Rep. 2019;19(6):5177–5184. doi:10.3892/mmr.2019.10179. 31059036

[25] Zuo L, Kuo W-T, Turner JR. Tight junctions as targets and effectors of mucosal immune homeostasis. Cell Mol Gastroenterol Hepatol. 2020;10(2):327–340. doi:10.1016/j.jcmgh.2020.04.001. 32304780 PMC7326733

[26] Oliveira SS, Morgado-Díaz JA. Claudins: multifunctional players in epithelial tight junctions and their role in cancer. Cell Mol Life Sci. 2007;64(1):17–28. doi:10.1007/s00018-006-6314-1. 17115119 PMC11136020

[27] Valle BL, Morin PJ. Chapter 13 – claudins in cancer biology. In: Yu AS L editor. Current topics in membranes. Academic Press, United States; 2010. p. 293–333.

[28] Turksen K, Troy T-C. Junctions gone bad: claudins and loss of the barrier in cancer. Biochim Biophys Acta. 2011;1816(1):73–79. doi:10.1016/j.bbcan.2011.04.001. 21515339

[29] Osanai M, Takasawa A, Murata M, Sawada N. Claudins in cancer: bench to bedside. Pflugers Arch. 2017;469(1):55–67. doi:10.1007/s00424-016-1877-7. 27624415

[30] Tabariès S, Siegel PM. The role of claudins in cancer metastasis. Oncogene. 2017;36(9):1176–1190. doi:10.1038/onc.2016.289. 27524421

[31] Gowrikumar S, Singh AB, Dhawan P. Role of claudin proteins in regulating cancer stem cells and chemoresistance-potential implication in disease prognosis and therapy. Int J Mol Sci. 2019;21(1):53. doi:10.3390/ijms21010053. PMC6982342 31861759

[32] Bhat AA, Syed N, Therachiyil L, Nisar S, Hashem S, Macha MA, Yadav SK, Krishnankutty R, Muralitharan S, Al-Naemi H, et al. Claudin-1, a double-edged sword in cancer. Int J Mol Sci. 2020;21(2):569. Available from: 10.3390/ijms21020569. PMC7013445 31952355

[33] Menard C, Pfau ML, Hodes GE, Kana V, Wang VX, Bouchard S, Takahashi A, Flanigan ME, Aleyasin H, LeClair KB, et al. Social stress induces neurovascular pathology promoting depression. Nat Neurosci. 2017;20(12):1752–1760. doi:10.1038/s41593-017-0010-3. 29184215 PMC5726568

[34] Nishiura K, Ichikawa-Tomikawa N, Sugimoto K, Kunii Y, Kashiwagi K, Tanaka M, Yokoyama Y, Hino M, Sugino T, Yabe H, et al. PKA activation and endothelial claudin-5 breakdown in the schizophrenic prefrontal cortex. Oncotarget. 2017;8(55):93382–93391. doi:10.18632/oncotarget.21850. 29212157 PMC5706803

[35] Greene C, Campbell M. Tight junction modulation of the blood brain barrier: CNS delivery of small molecules. Tissue Barriers. 2016;4(1):e1138017. doi:10.1080/21688370.2015.1138017. 27141420 PMC4836485

[36] Yamazaki Y, Shinohara M, Shinohara M, Yamazaki A, Murray ME, Liesinger AM, Heckman MG, Lesser ER, Parisi JE, Petersen RC, et al. Selective loss of cortical endothelial tight junction proteins during Alzheimer’s disease progression. Brain. 2019;142(4):1077–1092. doi:10.1093/brain/awz011. 30770921 PMC6439325

[37] Niu J, Tsai H-H, Hoi KK, Huang N, Yu G, Kim K, Baranzini SE, Xiao L, Chan JR, Fancy SPJ. Aberrant oligodendroglial-vascular interactions disrupt the blood-brain barrier, triggering CNS inflammation. Nat Neurosci. 2019;22(5):709–718. doi:10.1038/s41593-019-0369-4. 30988524 PMC6486410

[38] Dudek KA, Dion-Albert L, Lebel M, LeClair K, Labrecque S, Tuck E, Ferrer Perez C, Golden SA, Tamminga C, Turecki G, et al. Molecular adaptations of the blood-brain barrier promote stress resilience vs. Depression Proc Natl Acad Sci U S A. 2020;117(6):3326–3336. doi:10.1073/pnas.1914655117. 31974313 PMC7022213

[39] Greene C, Hanley N, Campbell M. Blood-brain barrier associated tight junction disruption is a hallmark feature of major psychiatric disorders. Transl Psychiatry. 2020;10(1):373. doi:10.1038/s41398-020-01054-3. 33139732 PMC7606459

[40] Greene C, Hanley N, Campbell M. Claudin-5: gatekeeper of neurological function. Fluids Barriers CNS. 2019;16(1):3. doi:10.1186/s12987-019-0123-z. 30691500 PMC6350359

[41] Chiba H, Ichikawa-Tomikawa N, Imura T, Sugimoto K. The region-selective regulation of endothelial claudin-5 expression and signaling in brain health and disorders. J Cell Physiol. 2021. Available from 10.1002/jcp.30357 33694168

[42] Spadaro D, Le S, Laroche T, Mean I, Jond L, Yan J, Citi S. Tension-dependent stretching activates ZO-1 to control the junctional localization of its interactors. Curr Biol. 2017;27(3783–3795.e8):3783–3795.e8. doi:10.1016/j.cub.2017.11.014. 29199076

[43] Zenker J, White MD, Gasnier M, Alvarez YD, Lim HYG, Bissiere S, Biro M, Plachta N. Expanding actin rings zipper the mouse embryo for blastocyst formation. Cell. 2018;173(3):776–791.e17. doi:10.1016/j.cell.2018.02.035. 29576449

[44] Bendriem RM, Singh S, Aleem AA, Antonetti DA, Ross ME. Tight junction protein occludin regulates progenitor self-renewal and survival in developing cortex. Elife. 2019;8:664. doi:10.7554/eLife.49376. PMC6890460 31794381

[45] Beutel O, Maraspini R, Pombo-García K, Martin-Lemaitre C, Honigmann A. Phase separation of zonula occludens proteins drives formation of tight junctions. Cell. 2019;179(4):923–936.e11. doi:10.1016/j.cell.2019.10.011. 31675499

[46] Kuo W-T, Shen L, Zuo L, Shashikanth N, Ong MLDM, Wu L, Zha J, Edelblum KL, Wang Y, Wang Y, et al. Inflammation-induced occludin downregulation limits epithelial apoptosis by suppressing caspase-3 expression. Gastroenterology. 2019;157(5):1323–1337. doi:10.1053/j.gastro.2019.07.058. 31401143 PMC6815722

[47] Otani T, Nguyen TP, Tokuda S, Sugihara K, Sugawara T, Furuse K, Miura T, Ebnet K, Furuse FM. Claudins and JAM-A coordinately regulate tight junction formation and epithelial polarity. J Cell Biol. 2019;218(10):3372–3396. doi:10.1083/jcb.201812157. 31467165 PMC6781433

[48] Schwayer C, Shamipour S, Pranjic-Ferscha K, Schauer A, Balda M, Tada M, Matter K, Heisenberg C-P. Mechanosensation of tight junctions depends on ZO-1 phase separation and flow. Cell. 2019;179(4):937–952.e18. doi:10.1016/j.cell.2019.10.006. 31675500

[49] Barnat M, Capizzi M, Aparicio E, Boluda S, Wennagel D, Kacher R, Kassem R, Lenoir S, Agasse F, Braz BY, et al. Huntington’s disease alters human neurodevelopment. Science. 2020;369(6505):787–793. doi:10.1126/science.aax3338. 32675289 PMC7859879

[50] Belardi B, Hamkins-Indik T, Harris AR, Kim J, Xu K, Fletcher DA, Weak A. Link with actin organizes tight junctions to control epithelial permeability. Dev Cell. 2020;54(792–804.e7):792–804.e7. doi:10.1016/j.devcel.2020.07.022. 32841596 PMC7530003

[51] Citi S. Cell Biology: Tight junctions as biomolecular condensates. Curr Biol. 2020;30(2):R83–6. doi:10.1016/j.cub.2019.11.060. 31962084

[52] Hartmann C, Schwietzer YA, Otani T, Furuse M, Ebnet K. Physiological functions of junctional adhesion molecules (JAMs) in tight junctions. Biochim Biophys Acta. 2020;1862(9):183299. doi:10.1016/j.bbamem.2020.183299. 32247783

[53] Lauko A, Mu Z, Gutmann DH, Naik UP, Lathia JD. Junctional adhesion molecules in cancer: a paradigm for the diverse functions of cell-cell interactions in tumor progression. Cancer Res. 2020;80(22):4878–4885. doi:10.1158/0008-5472.CAN-20-1829. 32816855 PMC7669553

[54] Otani T, Furuse M. Tight junction structure and function revisited. Trends Cell Biol. 2020;30(10):805–817. doi:10.1016/j.tcb.2020.08.004. 32891490

[55] Zhou T, Lu Y, Xu C, Wang R, Zhang L, Lu P. Occludin protects secretory cells from ER stress by facilitating SNARE-dependent apical protein exocytosis. Proc Natl Acad Sci U S A. 2020;117(9):4758–4769. doi:10.1073/pnas.1909731117. 32051248 PMC7060669

[56] Kubota H, Chiba H, Takakuwa Y, Osanai M, Tobioka H, Kohama G, Mori M, Retinoid SN. X receptor alpha and retinoic acid receptor gamma mediate expression of genes encoding tight-junction proteins and barrier function in F9 cells during visceral endodermal differentiation. Exp Cell Res. 2001;263(1):163–172. doi:10.1006/excr.2000.5113. 11161715

[57] Chiba H, Gotoh T, Kojima T, Satohisa S, Kikuchi K, Osanai M, Sawada N. Hepatocyte nuclear factor (HNF)-4alpha triggers formation of functional tight junctions and establishment of polarized epithelial morphology in F9 embryonal carcinoma cells. Exp Cell Res. 2003;286(2):288–297. doi:10.1016/S0014-4827(03)00116-2. 12749857

[58] Satohisa S, Chiba H, Osanai M, Ohno S, Kojima T, Saito T, Sawada N. Behavior of tight-junction, adherens-junction and cell polarity proteins during HNF-4alpha-induced epithelial polarization. Exp Cell Res. 2005;310(1):66–78. doi:10.1016/j.yexcr.2005.06.025. 16098509

[59] Chiba H, Sakai N, Murata M, Osanai M, Ninomiya T, Kojima T, Sawada N. The nuclear receptor hepatocyte nuclear factor 4alpha acts as a morphogen to induce the formation of microvilli. J Cell Biol. 2006;175(6):971–980. doi:10.1083/jcb.200608012. 17178913 PMC2064706

[60] Sugimoto K, Ichikawa-Tomikawa N, Satohisa S, Akashi Y, Kanai R, Saito T, Sawada N, Chiba H. The tight-junction protein claudin-6 induces epithelial differentiation from mouse F9 and embryonic stem cells. PLoS One. 2013;8(10):e75106. doi:10.1371/journal.pone.0075106. 24116027 PMC3792957

[61] Sugimoto K, Ichikawa-Tomikawa N, Kashiwagi K, Endo C, Tanaka S, Sawada N, Watabe T, Higashi T, Chiba H. Cell adhesion signals regulate the nuclear receptor activity. Proc Natl Acad Sci U S A. 2019;116(49):24600–24609. doi:10.1073/pnas.1913346116. 31740618 PMC6900646

[62] Li C-F, Chen J-Y, Ho Y-H, Hsu W-H, Wu L-C, Lan H-Y, Hsu D-S-S, Tai S-K, Chang Y-C, Yang M-H. Snail-induced claudin-11 prompts collective migration for tumour progression. Nat Cell Biol. 2019;21(2):251–262. doi:10.1038/s41556-018-0268-z. 30664792

[63] Singh AB, Sharma A, Dhawan P. Claudin-1 expression confers resistance to anoikis in colon cancer cells in a Src-dependent manner. Carcinogenesis. 2012;33(12):2538–2547. doi:10.1093/carcin/bgs275. 22941059 PMC3510734

[64] Huang H, Li L, Wu C, Schibli D, Colwill K, Ma S, Li C, Roy P, Ho K, Songyang Z, et al. Defining the specificity space of the human SRC homology 2 domain. Mol Cell Proteomics. 2008;7(4):768–784. doi:10.1074/mcp.M700312-MCP200. 17956856

[65] Filippakopoulos P, Müller S, Knapp S. SH2 domains: modulators of nonreceptor tyrosine kinase activity. Curr Opin Struct Biol. 2009;19(6):643–649. doi:10.1016/j.sbi.2009.10.001. 19926274 PMC2791838

[66] Liu BA, Engelmann BW, Jablonowski K, Higginbotham K, Stergachis AB, Nash PD. SRC homology 2 domain binding sites in insulin, IGF-1 and FGF receptor mediated signaling networks reveal an extensive potential interactome. Cell Commun Signal. 2012;10(1):27. doi:10.1186/1478-811X-10-27. 22974441 PMC3514216

[67] Mayer BJ. The discovery of modular binding domains: building blocks of cell signalling. Nat Rev Mol Cell Biol. 2015;16(11):691–698. doi:10.1038/nrm4068. 26420231

[68] Shimobaba S, Taga S, Akizuki R, Hichino A, Endo S, Matsunaga T, Watanabe R, Yamaguchi M, Yamazaki Y, Sugatani J, et al. Claudin-18 inhibits cell proliferation and motility mediated by inhibition of phosphorylation of PDK1 and Akt in human lung adenocarcinoma A549 cells. Biochim Biophys Acta. 2016;1863(6):1170–1178. doi:10.1016/j.bbamcr.2016.02.015. 26919807

[69] Luo J, Chimge N-O, Zhou B, Flodby P, Castaldi A, Firth AL, Liu Y, Wang H, Yang C, Marconett CN, et al. CLDN18.1 attenuates malignancy and related signaling pathways of lung adenocarcinoma in vivo and in vitro. Int J Cancer. 2018;143(12):3169–3180. doi:10.1002/ijc.31734. 30325015 PMC6263834

[70] Brunton VG, Irj M, Frame MC. Cell adhesion receptors, tyrosine kinases and actin modulators: a complex three-way circuitry. Biochim Biophys Acta. 2004;1692(2–3):121–144. doi:10.1016/j.bbamcr.2004.04.010. 15246683

[71] McLachlan RW, Kraemer A, Helwani FM, Kovacs EM, Yap AS, Mostov K. E-cadherin adhesion activates c-Src signaling at cell-cell contacts. Mol Biol Cell. 2007;18(8):3214–3223. doi:10.1091/mbc.e06-12-1154. 17553930 PMC1949350

[72] Cabodi S, Di Stefano P, Leal M, Del Pc TA, Bisaro B, Morello V, Damiano L, Aramu S, Repetto D, Tornillo G, et al. Integrins and signal transduction. Adv Exp Med Biol. 2010;674:43–54. 20549939 10.1007/978-1-4419-6066-5_5

[73] Canel M, Serrels A, Frame MC, Brunton VG. E-cadherin-integrin crosstalk in cancer invasion and metastasis. J Cell Sci. 2013;126(2):393–401. doi:10.1242/jcs.100115. 23525005

[74] Xiao R, Xi X-D, Chen Z, Chen S-J S-J, Meng G. Structural framework of c-Src activation by integrin β3. Blood. 2013;121(4):700–706. doi:10.1182/blood-2012-07-440644. 23169783

[75] Ochs K, Málaga-Trillo E. Common themes in PrP signaling: the Src remains the same. Front Cell Dev Biol. 2014;2:63. doi:10.3389/fcell.2014.00063. 25364767 PMC4211543

[76] Hamidi H, Ivaska J. Every step of the way: integrins in cancer progression and metastasis. Nat Rev Cancer. 2018;18(9):533–548. doi:10.1038/s41568-018-0038-z. 30002479 PMC6629548

[77] Witherden DA, Verdino P, Rieder SE, Garijo O, Mills RE, Teyton L, Fischer WH, Wilson IA, Havran WL. The junctional adhesion molecule JAML is a costimulatory receptor for epithelial gammadelta T cell activation. Science. 2010;329(5996):1205–1210. doi:10.1126/science.1192698. 20813954 PMC2943937

[78] Yaffe MB. MAGUK SH3 domains–swapped and stranded by their kinases? Structure. 2002;10(1):3–5. doi:10.1016/S0969-2126(01)00705-5. 11796103

[79] Lim WA, Pawson T. Phosphotyrosine signaling: evolving a new cellular communication system. Cell. 2010;142(5):661–667. doi:10.1016/j.cell.2010.08.023. 20813250 PMC2950826

[80] Liu BA, Nash PD. Evolution of SH2 domains and phosphotyrosine signalling networks. Philos Trans R Soc Lond B Biol Sci. 2012;367(1602):2556–2573. doi:10.1098/rstb.2012.0107. 22889907 PMC3415846

[81] Wagner MJ, Stacey MM, Liu BA, Pawson T. Molecular mechanisms of SH2- and PTB-domain-containing proteins in receptor tyrosine kinase signaling. Cold Spring Harb Perspect Biol. 2013;5(12):a008987. doi:10.1101/cshperspect.a008987. 24296166 PMC3839611

[82] Kojima M, Sugimoto K, Tanaka M, Endo Y, Kato H, Honda T, Furukawa S, Nishiyama H, Watanabe T, Soeda S, et al. Prognostic significance of aberrant claudin-6 expression in endometrial cancer. Cancers. 2020;12(10):2748. Available from: 10.3390/cancers12102748. PMC7656298 32987797

[83] Kojima M, Sugimoto K, Kobayashi M, Ichikawa-Tomikawa N, Kashiwagi K, Watanabe T, Soeda S, Fujimori K, Chiba H. Aberrant claudin-6-adhesion signaling promotes endometrial cancer progression via estrogen receptor α. Mol Cancer Res [*in press*; Available from]. 2021. https://mcr.aacrjournals.org/content/early/2021/03/16/1541-7786.MCR-20-0835 10.1158/1541-7786.MCR-20-0835 33727343

[84] Lang F, Böhmer C, Palmada M, Seebohm G, Strutz-Seebohm N, Vallon V. (Patho)physiological significance of the serum- and glucocorticoid-inducible kinase isoforms. Physiol Rev. 2006;86(4):1151–1178. doi:10.1152/physrev.00050.2005. 17015487

[85] Hagen SJ, Ang L-H, Zheng Y, Karahan SN, Wu J, Wang YE, Caron TJ, Gad AP, Muthupalani S, Fox JG. Loss of tight junction protein claudin 18 promotes progressive neoplasia development in mouse stomach. Gastroenterology. 2018;155(6):1852–1867. doi:10.1053/j.gastro.2018.08.041. 30195448 PMC6613545

[86] Zhou B, Flodby P, Luo J, Castillo DR, Liu Y, Yu F-X, McConnell A, Varghese B, Li G, Chimge N-O, et al. Claudin-18-mediated YAP activity regulates lung stem and progenitor cell homeostasis and tumorigenesis. J Clin Invest. 2018;128(3):970–984. doi:10.1172/JCI90429. 29400695 PMC5824875

[87] Suzuki K, Sentani K, Tanaka H, Yano T, Suzuki K, Oshima M, Yasui W, Tamura A, Tsukita S. Deficiency of stomach-type claudin-18 in mice induces gastric tumor formation independent of H pylori infection. Cell Mol Gastroenterol Hepatol. 2019;8(1):119–142. doi:10.1016/j.jcmgh.2019.03.003. 30910700 PMC6554658

[88] Paquet-Fifield S, Koh SL, Cheng L, Beyit LM, Shembrey C, Mølck C, Behrenbruch C, Papin M, Gironella M, Guelfi S, et al. Tight junction protein claudin-2 promotes self-renewal of human colorectal cancer stem-like cells. Cancer Res. 2018;78(11):2925–2938. doi:10.1158/0008-5472.CAN-17-1869. 29510994

